# Modes of Human T Cell Leukemia Virus Type 1 Transmission, Replication and Persistence

**DOI:** 10.3390/v7072793

**Published:** 2015-07-07

**Authors:** Alexandre Carpentier, Pierre-Yves Barez, Malik Hamaidia, Hélène Gazon, Alix de Brogniez, Srikanth Perike, Nicolas Gillet, Luc Willems

**Affiliations:** Molecular and Cellular Epigenetics (GIGA) and Molecular Biology (Gembloux Agro-Bio Tech), University of Liège (ULg), 4000 Liège, Belgium; E-Mails: a.carpentier@doct.ulg.ac.be (A.C.); py.barez@doct.ulg.ac.be (P.-Y.B.); mhamaidia@ulg.ac.be (M.H.); helene.gazon@ulg.ac.be (H.G.); alix.debrogniez@ulg.ac.be (A.B.); Srikanthperike@gmail.com (S.P.); n.gillet@ulg.ac.be (N.G.)

**Keywords:** HTLV-1, viral replication, viral persistence, Tax, HBZ

## Abstract

Human T-cell leukemia virus type 1 (HTLV-1) is a retrovirus that causes cancer (Adult T cell Leukemia, ATL) and a spectrum of inflammatory diseases (mainly HTLV-associated myelopathy—tropical spastic paraparesis, HAM/TSP). Since virions are particularly unstable, HTLV-1 transmission primarily occurs by transfer of a cell carrying an integrated provirus. After transcription, the viral genomic RNA undergoes reverse transcription and integration into the chromosomal DNA of a cell from the newly infected host. The virus then replicates by either one of two modes: (i) an infectious cycle by virus budding and infection of new targets and (ii) mitotic division of cells harboring an integrated provirus. HTLV-1 replication initiates a series of mechanisms in the host including antiviral immunity and checkpoint control of cell proliferation. HTLV-1 has elaborated strategies to counteract these defense mechanisms allowing continuous persistence in humans.

## 1. Introduction

HTLV-1 infects approximately 5–10 million people worldwide mainly in subtropical areas [1]. In the vast majority of cases, HTLV-1 infection remains clinically silent. Among asymptomatic carriers, 3% to 5% will develop a leukemia/lymphoma (ATL) or a neurodegenerative disease (HAM/TSP) after long latent periods (40–60 years) [2]. ATL results from proliferation and accumulation of infected cells carrying an integrated proviral genome (here referred to as clones). HAM/TSP is associated with invasion of the central nervous system by infected cells, antiviral immunity, cytokine burst, and inflammation. Main clinical symptoms of HAM/TSP are urinary failures and paralysis of lower legs. Why infected subjects develop either ATL or HAM/TSP is currently unknown. There is no efficient treatment for HAM/TSP, except palliative attenuation of inflammation with corticosteroids. The leukemic form of ATL is initially responsive to general chemotherapy (CHOP) but almost invariably relapses after a few months. An antiviral therapy based on AZT combined with interferon yields 50% survival at five years [3]. Another type of treatment includes hematopoietic stem cell transplantation that yields, if successful, the best long-term survival rates [4,5,6]. An anti-CCR4 antibody is now in clinical use in Japan [7,8] and other promising approaches such as valproic acid are currently being investigated [8,9].

The HTLV-1 genome contains essential structural and enzymatic genes (Gag, Pro, Pol and Env) shared by all retroviral family members (reviewed by [10]). As a deltaretrovirus, HTLV-1 also encodes a series of accessory and regulatory proteins. Among these, the Tax oncoprotein and HTLV-1 basic leucine zipper factor (HBZ) play pivotal roles in the viral life cycle [11]. Here, we describe how these factors subvert cellular pathways to allow viral transmission, persistence, and replication.

## 2. Current Model of HTLV-1 Replication

**Figure 1:**
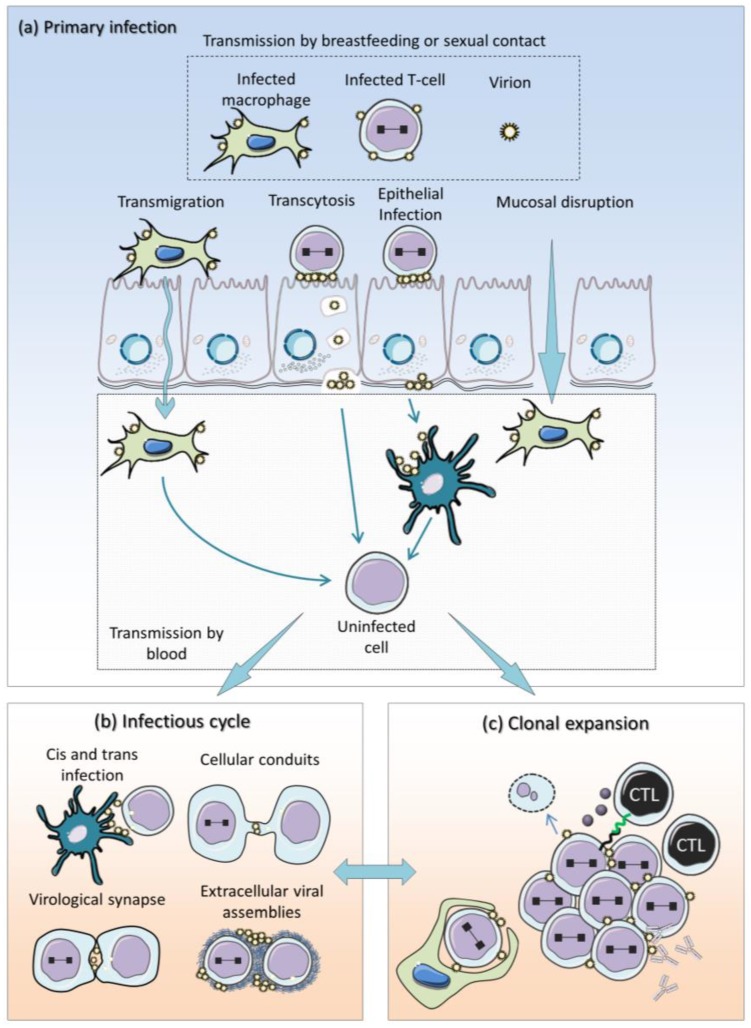
Model of HTLV-1 replication (**a**) HTLV-1 transmission occurs by breastfeeding, sexual intercourse, or blood transfusion. Except for blood transfer, initial infection requires crossing of the mucosal barrier by several mechanisms: (i) transmigration of HTLV-1 infected macrophages, (ii) transcytosis of viral particles, (iii) release of newly produced virions from the basal surface of infected epithelial cell, (iv) bypass of HTLV-1 infected cells through a damaged mucosa. HTLV-1 can then infect mucosal immune cells directly (cis-infection) or via antigen-presenting cells (APCs); (**b**) APCs can either become infected or transfer membrane-bound extracellular virions to T-cells (trans-infection). Cell-to-cell transfer of virions involves different non-exclusive mechanisms: a virological synapse, cellular conduits, or extracellular viral assemblies. Infection of resident cells occurs either in the mucosa or in secondary lymphoid organs. Soon after primary infection, HTLV-1 replicates by cell-to-cell infection (*i.e.*, the infectious cycle) or (**c**) by mitotic division of a cell containing an integrated provirus (clonal expansion). Since an antiviral immune response is quickly initiated, the efficacy of the infectious cycle is severely dampened down soon after infection.

HTLV-1 predominantly infects CD4+ T cells but also targets other cell types such as CD8+ T and B lymphocytes, dendritic cells (DCs), monocytes, and macrophages [12,13,14]. This pleiotropic pattern is permitted by the presence of membrane-associated receptors that interact with the viral envelope allowing efficient binding and entry. These include heparan sulfate proteoglycans (HSPGs), the glucose transporter 1 (GLUT-1) and neuropilin-1 (NRP-1) [15,16,17,18]. The mechanisms of receptor binding and virus entry have been reviewed elsewhere [19,20,21]. A number of studies have shown that cell-free infection is poorly efficient compared to cell-to-cell virus transfer (about 10,000 fold) [22,23], suggesting that HTLV-1 spread *in vivo* relies more on a cellular intermediate than on the virion itself. Whatever the route of infection used, the initial contact with HTLV-1 mainly occurs via breast feeding, sexual intercourse, and blood transfusion [24]. Except when contamination occurs by blood transfer, initial infection first requires interaction with oral, gastrointestinal, or cervical mucosa. Crossing of the mucosal barrier occurs by different mechanisms as schematized on Figure 1a. Although not formally demonstrated yet, HTLV-1 infected macrophages could transmigrate through an intact epithelium as observed for human immunodeficiency virus (HIV) [25,26]. Viral particles produced by HTLV-1 infected T-cells have been shown to cross the epithelium by transcytosis, *i.e.*, the transit of a virion incorporated into a vesicle from the apical to the basal surface of an epithelial cell [26,27]. Alternatively, HTLV-1 can also infect an epithelial cell and produce new virions that are then released from the basal surface [28]. Finally, HTLV-1 infected cells can directly bypass a disrupted mucosa [28]. 

Having crossed the epithelial barrier, HTLV-1 infects mucosal immune cells directly or via APCs such as DCs or macrophages. APCs can either undergo infection or transfer membrane bound extracellular virions to uninfected T-cells (trans-infection) [14]. Cell-to-cell transfer of HTLV-1 virions then potentially involves several non-exclusive mechanisms (reviewed in [28]): a virological synapse [29,30,31], cellular conduits [32], or extracellular viral assemblies [33,34]. Infection of resident cells occurs either in the mucosa or in secondary lymphoid organs. 

Soon after primary infection, HTLV-1 attempts to expand by colonizing new targets by cell-to-cell transfer, reverse transcription of the viral RNA, integration of the provirus into the chromosome, expression of viral proteins and budding of new virions (the infectious cycle; Figure 1b). Another mode of replication involves mitotic division of a cell containing an integrated provirus (clonal expansion; Figure 1c). Recently, host restriction factors such as SAMHD1, APOBEC3 and miR-28-3p have been shown to limit HTLV-1 infection [35,36,37]. Since an antiviral immune response is also quickly initiated, the efficacy of the infectious cycle is severely attenuated soon after infection, although likely not completely abrogated later on. On the other side, clonal expansion and cell proliferation also require expression of viral factors such as Tax [38,39]. Survival of infected progeny cells therefore requires silencing of viral expression before immune-mediated destruction. This model is consistent with the following observations: (i) to block HTLV-1 infection, reverse transcriptase inhibitors (RTIs) must be administrated simultaneously with viral inoculation [40]; (ii) when used alone, RTIs do not reduce the proviral load in HTLV-1 infected subjects [41,42]; (iii) sustained T-cell proliferation in patients correlates with Tax expression [43], extending previous studies in BLV-infected animal models [44]; (iv) compared to HIV, the HTLV-1 genome undergoes limited variability [45], suggesting a replication mode by cellular DNA polymerase rather than by viral reverse transcriptase; (v) sequential high-throughput sequencing of proviral integration sites reveal a high clonal stability over years [46]. In this context, our recent study in BLV-infected cows also showed that most clones generated during primary infection are destroyed and replaced by others undergoing expansion [47].

Taken together, these data support a model of viral replication by cell-to-cell contact at the early stages of infection, followed by a sustained clonal proliferation counterbalancing the host immune response. Repetitive cycles of viral expression followed by transcriptional silencing continuously challenges the immune response thereby initiating inflammation and ultimately leading to HAM/TSP. By favoring emergence of sporadic mutations in the cell genome, unrestrained proliferation also paves the way to malignant transformation and development of ATL [43].

## 3. Tax and HBZ Are Two Main Drivers of Viral Replication

**Figure 2:**
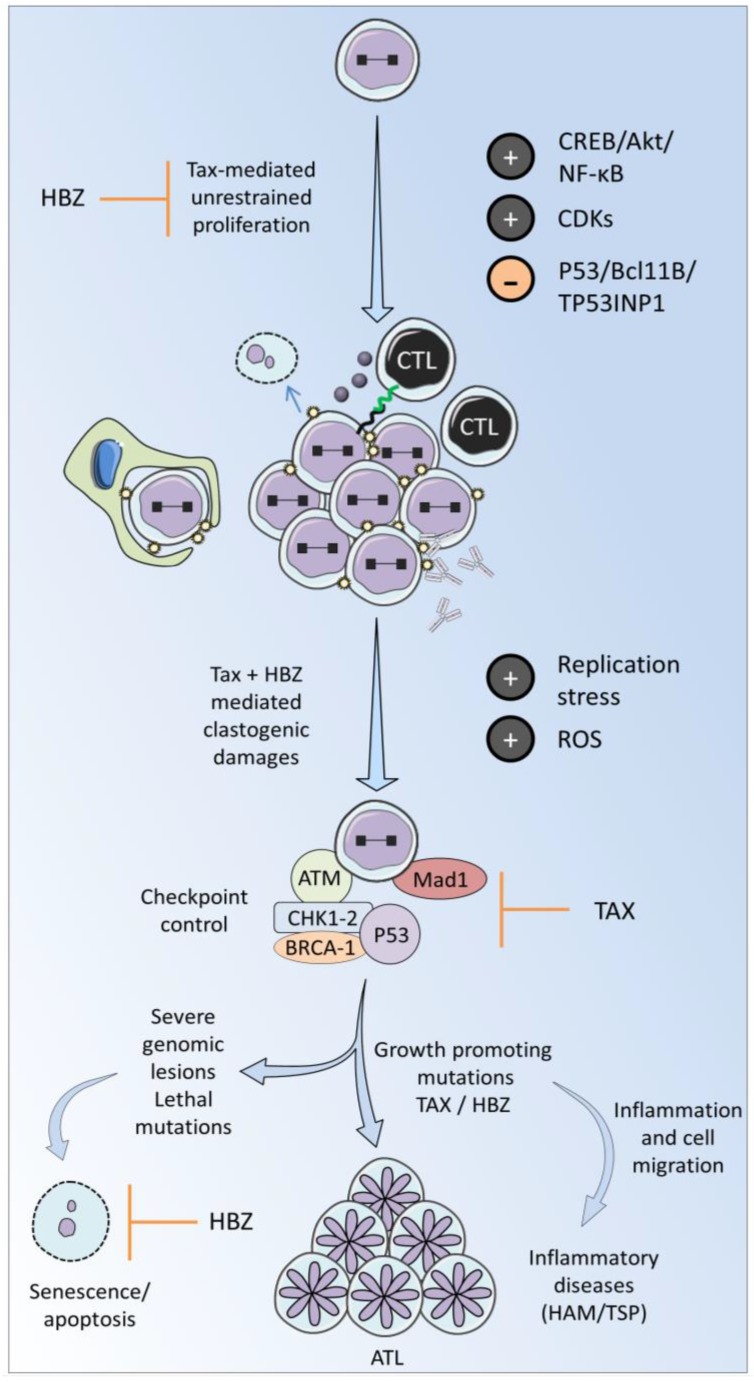
Tax and HBZ promote proliferation and persistence of the infected cell. Tax activates survival pathways (CREB/Akt/NFkB), promotes mitosis (CDKs), and inhibits tumor suppressors (p53, TP53INP1, Bcl11B). Tax-mediated growth-promoting activities are counteracted by HBZ, mitigating unrestrained proliferation. The host immune response further controls infected cell proliferation. Tax-induced proliferation creates replicative stress and generates reactive oxygen species (ROS). Tax interacts with the mitotic checkpoint control protein Mad1 thereby inducing clastogenic damage. Tax attenuates the DNA damage response (DDR) induced by unscheduled cell proliferation. Inhibition of the DDR allows cells to accumulate DNA lesions and stabilize mutations. If uncontrolled by senescence or cell death mechanisms, growth-promoting mutations pave the way to disease development.

According to currently most accepted model, Tax and HBZ are believed to have the highest impact on viral replication and cell transformation, besides other components required to synthesize the viral particle. The modes of action of Tax and HBZ are remarkably pleiotropic and involve a variety of cell signaling pathways (CREB, NFkB and AKT; Figure 2). Tax inhibits tumor suppressors (p53, Bcl11B and TP53INP1 [48,49,50]) and activates cyclin-dependent kinases (CDKs) [51], both of these mechanisms leading to accelerated cell proliferation. In parallel, Tax attenuates the Mad1 spindle assembly checkpoint protein, induces genomic lesions and interferes with DNA repair thereby promoting aneuploidy [39,52,53]. Experimental evidence also shows that Tax drives tumor formation in transgenic mouse models, supporting its oncogenic potential [54,55,56]. Tax also induces genomic instability [39,57], generating somatic alterations [58] and promoting cell growth. However, expression of Tax alone fails to systematically immortalize human primary T cells [59], suggesting the involvement of other viral or cellular components. In particular, driver mutations affecting the CCR4 chemokine receptor have been identified in ~25% of ATL cases [60,61]. In about 50% of ATL cases, Tax is either inactivated by genetic mutation or transcriptionally silenced by hyper-methylation or deletion of the 5′-LTR [62,63,64,65]. Because of the strong immunogenicity of the Tax protein, it is possible that these mechanisms confer a selective advantage to HTLV-1-transformed T cells [66,67,68,69]. In comparison, HBZ triggers a less efficient immunity that is compatible with permanent expression throughout HTLV-1 infection [70,71]. Later in leukemogenesis, cell growth can thereby become independent of Tax and be promoted by HBZ. Indeed, HBZ is constitutively expressed throughout HTLV-1 infection [72,73], counteracts Tax-mediated viral and cellular pathways modulation (such as NF-κB, Akt and CREB) and stimulates cell proliferation [69,74] via apoptosis/senescence inhibition and cell cycle modulation [69,74]. This simplified model thus hypothesizes that Tax initiates transformation while HBZ is required to maintain the transformed phenotype if Tax expression is silenced [75]. Clinical data indicating that Tax mRNA expression allows estimating the risk of HAM/TSP development and that HBZ positively correlates with the severity of symptoms further supports a role of Tax and HBZ in pathogenesis [76,77].

### 3.1. Tax and HBZ Exert Opposite Functions in Signaling Pathways

Almost systematically, the activities of Tax on a series of cellular pathways are balanced by HBZ. 

#### 3.1.1. NF-κB 

By controlling T lymphocyte activation and proliferation in response to diverse immune stimuli (such as antigens, cytokines or microbial components), the NF-κB pathway is a key player in regulation of immunity and inflammation [78]. HTLV-1 Tax activates the IKK complex through IKKγ/NEMO binding. Tax requires CADM1/TSLC1 for inactivation of the NF-kappaB inhibitor A20 and constitutive NF-κB signaling [79]. The subsequent translocation of p50/p65 complex into the nucleus activates transcription of NF-κB responsive genes [78,80]. Activation of the canonical NF-κB pathway by Tax requires IL17RB signaling [81]. On the other hand, Tax stimulates IKKα-dependent processing of p100 into p52 [78,80]. Tax also hijacks the cellular ubiquitin machinery to activate ubiquitin-dependent kinases and NF-κB signaling (reviewed in [82]). Tax thereby induces expression of a variety of growth promoting cytokines (such as IL-1, IL-6, TNF, and EGF [83,84]). Tax also upregulates antiapoptotic proteins: caspase-8 inhibitory protein c-FLIP [85,86] and members of the Bcl-2 family (Bcl-2, Bcl-xL, Mcl-1 and Blf-1) [87,88,89,90]. By activating the NF-κB pathway, Tax thus favors proliferation and survival of HTLV-1-infected T cells. On the contrary, HBZ suppresses the canonical NF-κB signaling pathway by inhibiting the activity of the RelA/p65 complex and thus mitigates excessive activation of NF-κB by Tax [91]. NF-κB activation by Tax is associated with an upregulation of p21^WAF1/CIP1^ and p27^KIP1^, leading to cellular senescence [92,93]. In HeLa cells, HBZ prevents Tax-induced senescence through down-regulation of NF-κB [92,94]. 

#### 3.1.2. Akt

Tax promotes cell proliferation and survival through activator protein-1 (AP-1) and the phosphatidylinositol 3-kinase (PI3K)/Akt pathway [95]. Inhibition of Akt in HTLV-1-transformed cells decreases phosphorylated Bad and induces caspase-dependent apoptosis [96]. Stimulation of PI3K/Akt by Tax activates HiF-1 (hypoxia-inducible factor 1) [97], reduces expression of proapoptotic Bim and Bid and promotes IL-2 independent growth [98] and finally increases Bcl3 whose expression is associated with the growth of infected cells [99]. HBZ inhibits Tax-dependent activation of the PI3K/Akt pathway and downstream anti-apoptotic properties [100]. HBZ suppresses apoptosis by attenuating the function of FOXO3a and altering its localization [101]. Besides, the interaction of HBZ with AP-1 factors (cJun, JunB or MafB) results in the inhibition of their transcriptional activities via several mechanisms, such as sequestration into nuclear bodies or proteasomal degradation, and prevents the subsequent activation of AP-1 regulated genes [102,103,104,105,106].

#### 3.1.3. CREB

Tax activates 5′-LTR-directed transcription by interacting with CREB, modulating its phosphorylation at Ser133 and connecting the histone acetyltransferase CBP (CREB-binding protein/p300) [107]. The ability of Tax to activate transcription via CREB is required to protect murine fibroblasts from serum-depletion-induced apoptosis. [108,109,110]. Tax modifies the phosphorylation state of CREB (i) by activating the upstream Akt kinase [111,112] or (ii) by decreasing the expression of PTEN phosphatase which is required for CREB dephosphorylation at Ser133 in the nucleus [111,113]. 

HBZ represses viral transcription through interaction with the bZIP domain of CREB proteins and prevents their binding to the viral CRE elements [114,115]. HBZ interacts with the KIX domain of p300/CBP, competing for Tax binding and inhibiting the association of co-activators with the viral promoter [116]. HBZ modulates the occupancy of KIX domains of p300/CBP by modulating the activity of transcription factors, thereby influencing subsequent gene expression [117].

#### 3.1.4. Wnt 

Tax interacts with DAPLE (dishevelled-associating protein with a high frequency of leucine residues) to activate the canonical Wnt pathway. HBZ can suppress this activation by inhibiting DNA binding of TCF-1/LEF-1 transcription factors. On the other side, HBZ promotes transcription of WnT 5a, a key protein of the non-canonical Wnt pathway, by enhancing its promoter activity through transforming growth factor-beta (TGF-beta). Knockdown of Wnt5a represses proliferation and migration of ATL cells, pointing out the role of this pathway in HTLV-1 infected cell growth [118]. 

#### 3.1.5. TGF-β/Smad 

Tax represses TGF-β 1 signaling (i) by blocking the association of Smad proteins with Smad-binding elements, (ii) through its interaction with CREB-binding protein/p300 and (iii) via c-jun activation [119,120,121]. HBZ counteracts this effect and also interacts with Smad2/3 to enhance TGF-beta/Smad transcriptional responses in a p300-dependent manner, improving transcription of different genes, such as the FOXP3 mediator of regulatory T cells [122].

#### 3.1.6. S Phase Entry and Cell Cycle Progression

Through interaction with cyclins and CDKs, Tax interferes with cell cycle progression by several mechanisms: (i) Tax stabilizes the cyclin D2/CDK4 complex and favors hyperphosphorylation of the retinoblastoma protein (Rb). Phosphorylated Rb frees E2F1 that activates transcription of genes required for G1/S transition; (ii) Tax represses cyclin-dependent kinases inhibitors (CKIs) such as members of INK4 family and KIP1; (iii) Tax interacts with and directs Rb to the proteasome for subsequent degradation; (iv) Tax activates the cyclin D1 transcription by enhancing p300 recruitment to the CRE site of cyclin D1 promoter through interaction with pCREB and TORC2 [123]. As a result, Tax favors S phase entry of HTLV-1 infected cells (reviewed in [51,75]). Tax also accelerates S phase progression by interaction with the replicative helicase (minichromosome maintenance complex, MCM2-7). Tax modulates the spatiotemporal program of replication origins through p300-dependent histone hyperacetylation, resulting in early firing of late replication origins. Tax also fires supplementary origins of replication accelerating S phase progression. This mechanism triggers replicative stress and genomic lesions, such as double strand breaks (DSBs) [39,57]. By modulating replication timing, Tax could also modulate the entire transcriptional landscape of infected cells. Indeed, the level of transcription at replication origins (ORC1 binding sites) correlates with replication timing [124].

In contrast to Tax, HBZ exerts a dual regulatory role in cell cycle progression. Indeed, HBZ interacts with CREB and inhibits transcription of cyclin D1 [125]. HBZ also binds activating transcription factor 3 (ATF3) that modulates expression of cell division cycle 2 (CDC2) and cyclin E2, thereby promoting proliferation of ATL cells [126]. Concomitantly, HBZ suppresses ATF3-induced p53 transcriptional activity. Moreover, the HBZ mRNA increases E2F1 gene transcription and promotes cell proliferation [69].

Together, this series of data on the signaling pathways illustrates the opposite functions of Tax and HBZ in finely tuned regulatory mechanisms of cell proliferation. 

### 3.2. Cellular Checkpoints Control Unscheduled Proliferation

Cellular checkpoints act as a failsafe barrier against unrestrained cellular proliferation. Tax subverts the G1 restriction and the spindle mitotic checkpoints. In G1, the tumor-suppressor protein p53 is the main factor that controls the checkpoint. Although approximately 50% of cancers harbor a mutation in p53, this mechanism only appears in a small percentage of ATL patients. Instead, p53 is functionally inactivated in leukemic and HTLV-1 transformed cells [127]. It remains incompletely understood how Tax inactivates p53: (i) Tax competes with p53 in binding with CBP, thereby repressing p53 trans-activating function [128]; (ii) NF-kappaB p65 subunit is critical for Tax-induced p53 inactivation [129]; (iii) repression of p53 transcriptional activity by Tax is independent of NF-kB and CBP [130]; p53 is invalidated by wild-type p53-induced phosphatase 1 (Wip1) [131,132]. ATL cells are characterized by loss of spindle assembly checkpoint function [133] and aneuploidy [134]. Tax binding to Mad1 perturbs the organization of the spindle assembly and results in multinucleated cells [52]. Moreover, the direct interaction between Tax and the anaphase-promoting complex APC Cdc20 also explains the mitotic abnormalities in HTLV-1 infected cells [135]. Tax promotion of supernumerary centrosomes through recruitment of Ran and Ran-binding protein-1 is another mechanism contributing to leukemia [136].

### 3.3. Response to DNA Damage 

By accelerating the replication-timing program, the Tax protein induces replicative stress and DSBs [39]. Tax expression generates reactive oxygen species (ROS) leading to oxidative and replication-dependent DSBs [53]. Tax-associated DNA damages activate several phosphoproteins of the DDR pathway (H2AX, ATM, CHK1-2, P53, BRCA1), which in turn arrest the cell cycle transiently or lead to apoptosis and senescence. In presence of DNA damaging agents (e.g., UV irradiation), Tax inhibits the DDR machinery by sequestrating key signaling pathway components [137,138,139,140,141,142,143,144]. Induction of genomic lesions and inhibition of the DDR leads to proliferation in presence of DNA mutations, potentially to leukemogenesis. 

HBZ induces DNA lesions through activation of miR-17 and miR-21 and downregulation of the DNA damage factor OBFC2A [145]. HBZ association with growth arrest and DNA damage gene 34 (GADD34) also deregulates the cellular responses to DNA damage [146].

### 3.4. DNA Repair Pathways

Besides modulating the DDR signaling pathway, Tax also directly interferes with the mechanisms of DNA repair. For example, Tax downregulates the expression of β-polymerase [147] and inhibits base excision repair (BER) [148]. Furthermore, Tax activates PCNA and interferes with nucleotide excision repair (NER) [149,150]. Tax decreases Ku80 gene transcription and interacts with Ku80 protein, interfering with non-homologous end joining (NHEJ) [151,152]. In Tax-expressing cells, DSBs are nevertheless preferentially repaired by error-prone NHEJ [153]. Another viral protein, p30, inhibits homologous recombination, shifting repair towards unfaithful pathways [154]. Whether HBZ also interferes with DNA damage repair mechanisms remains to be further clarified.

## 4. Conclusions

HTLV-1 persists and replicates by means of viral proteins, such as Tax and HBZ that finely tune cellular signaling pathways. Viral replication through the infectious and the mitotic routes requires viral expression and faces destruction by the host immune response. Expression of viral proteins creates genomic stress responsible for DNA lesions that initiate the DDR response. Imperfect repair of these errors stabilizes mutations that potentially drive oncogenesis.
